# Supplementary material for: Mental practice in isolation improves cervical joint position sense in patients with chronic neck pain: a randomized single-blind placebo trial
Source: PeerJ. 2019 Sep 12;7:e7681. doi: 10.7717/peerj.7681 (PMC6745187; doi:10.7717/peerj.7681)
Supplement: Supplemental Information 3 [file peerj-07-7681-s003.docx]

**Criterios de estilo y forma para completar el Protocolo de Evaluación de Investigación por parte del CEI**

1. El protocolo se rellenará utilizando como tipo de letra Calibrí 12, cuerpo, sin negrita.
2. Ningún punto del protocolo debe quedar vacío. Si algún punto no es aplicable al proyecto que se presenta debe hacerse constar como “no procede”.
3. Incluir una breve descripción de las pruebas de evaluación que van a utilizarse.
4. Los documentos como consentimientos se presentarán como anexos los cuales estarán nominados preferiblemente con “nombre” no con número. En el caso de estar nominados el protocolo será numerado con e nº1, el consentimiento informado el nº 2 y posteriormente el resto de los documentos que se anexen.
5. Existe un modelo básico de CI, con él no será necesario incluir una hoja informativa aparte pues toda la información que debe darse al sujeto de investigación está incluida. Se firmará por duplicado para que tanto el investigador como el sujeto de investigación tengan una copia.
6. De acuerdo con lo dispuesto en Reglamento de la Unión Europea 2016/679 de Protección de Datos de Carácter Personal (RGPD 2016/679), debe aparecer en el CI el nombre del fichero en el que se conservan los datos específicos de una investigación. Puede tener el nombre de la investigación o un número, pero es imprescindible que aparezca.
7. De la misma forma y según lo dispuesto en el reglamento mencionado, y, según el art. 13 RGPD debe informarse de:
   1. el nombre y forma de contacto del responsable final de la custodia de los datos.
   2. los datos de contacto del delegado de protección de datos (Castañeda Abogados);
   3. los fines del tratamiento a que se destinan los datos personales (prestación de asistencia sanitaria) y la base jurídica del tratamiento (consentimiento);
   4. las categorías de destinatarios de los datos personales (p.ej. personal externo, proveedores de sistemas);
   5. el plazo durante el cual se conservarán los datos personales (5 años desde la fecha del alta para datos de salud);
   6. la existencia del derecho a solicitar La Salle el acceso a los datos personales, y su rectificación o supresión, o la limitación de su tratamiento, o a oponerse al tratamiento, así como el
   7. derecho a la portabilidad de los datos;
   8. la existencia del derecho a retirar el consentimiento en cualquier momento;
   9. el derecho a presentar una reclamación ante la Agencia Española de Protección de Datos.
8. Cuando se realice la investigación en un centro externo será imprescindible una autorización de ese centro para la captación de la muestra, el uso de sus instalaciones sea de la naturaleza que sea así como incluir a una persona de referencia en dicho centro que facilite la labor del investigador; así como la suscripción de un contrato que regule el acceso a datos de carácter personal, si el mismo tuviera lugar por parte del centro.
9. En el caso de realizarse la investigación en un centro externo con el que se haya realizado un convenio de investigación debe aportarse la referencia a este convenio, fecha de firma y duración. De igual manera se incluirá en el proyecto a la persona de referencia del centro externo.
10. En el caso de que la investigación a evaluar se haga con un programa informático debe incluirse la consulta previa a la AEMPS sobre su consideración o no como producto sanitario. En el caso de utilizarse un producto sanitario debe incluirse el marcado CE con las indicaciones específicas de uso de dicho producto

PROTOCOLO PARA LA VALORACIÓN DE PROYECTOS CON SERES HUMANOS DEL COMITÉ DE ÉTICA DE INVESTIGACIÓN DEL CSEULS

| 1. Título de la investigación: Efectos de la Imaginería Motora y la Observación de Acciones sobre el control motor cervical.   Línea de investigación a la que se adscribe: Efectos motores de la movilización del sistema nervioso en un modelo de dolor inducido craneocervical  Responsable de la misma: Roy La Touche Arbizu |
| --- |
| 1. b del Investigador principal: (adjuntar CV) Roy La Touche Arbizu   Teléfono:  Email: roylatouche@lasallecampus.es |
| 1. Facultad/departamento del que depende el investigador principal.   Facultad de Ciencias de la Salud del Centro Superior de Estudios Universitarios La Salle, Departamento de Fisioterapia. |
| 1. Nombre y Apellidos del resto de investigadores (adjuntar CV)   Investigador 1: Ferran Cuenca Martínez  Teléfono:  Email: fecuen2@gmail.com  Investigador 2: Luis Suso Martí  Teléfono:  Email: Luis.suso@gmail.com |
| 1. Facultad/departamento del que dependen el resto de los investigadores.   Facultad de Ciencias de la Salud del Centro Superior de Estudios Universitarios La Salle, Departamento de Fisioterapia. |
| 1. Institución o entidad donde se llevará a cabo la investigación. Si es una institución externa al CSEULS indicar:   Laboratorio del Centro Superior de Estudios Universitarios La Salle.   - - Nombre y apellidos de la persona responsable   - Cargo   - Aspectos concretos de la investigación para los que autoriza   ¿Se presenta autorización del centro externo para la recogida de muestra u otros aspectos de la realización del proyecto?  Sí  No |
| 1. Entidad que financia el proyecto   Describir a continuación con qué medios económicos prevé realizar el proyecto, considerando también la posibilidad de no recibir la financiación solicitada  Los medios económicos irán a cargo de los investigadores, donde no habrá financiación económica del presente proyecto de investigación ergo, no procede.  Indique a qué convocatorias tiene previsto presentar el proyecto: no procede Elija un elemento. |
| 1. Fecha inicio:   Fecha finalización: |
| VALOR SOCIAL Y JUSTIFICACIÓN |
| 1. Antecedentes, estado del tema anterior a la investigación. (máximo 12 líneas)   La imaginería motora (IM) es la representación mental de un movimiento (1), mientras que la observación de acciones (OA) consiste en observar acciones específicas realizados por una tercera persona (2). Entre los efectos más estudiados están: mejora en el rendimiento deportivo (3), patologías neurológicas sobre todo en los últimos años (4-6), dolor crónico e inmovilizaciones (4).  Estas aplicaciones se deben a los conocimientos acerca de las áreas corticales que activa, demostrando influencia sobre el Sistema Nervioso Central (SNC) y producción de cambios en la plasticidad neural (7).  Ya se han realizado estudios sobre el efecto de IM y OA en sujetos sanos (4,8), en ambos miembros y en la región cervical, con incrementos significativos en la percepción de fatiga mental (9-11).  En algunas investigaciones se obtuvieron una disminución de la fuerza, tras las sesiones, en los grupos experimentales (9,11). A medio plazo, otros estudios, demostraron mayor aumento de fuerza muscular si ambas técnicas son combinadas (12-14). Para finalizar, existen estudios, aunque escasos, sobre los efectos en el control motor (15) y postural (16), obteniendo mejoras significativas. |
| 1. Justificación del proyecto (máximo 15 líneas)   Tanto la imaginería motora, como la observación de acciones, combinadas con el movimiento llevado a cabo de manera real, son herramientas en las que se han obtenido muy buenos resultados a lo largo de los diferentes estudios en distintas zonas corporales (3-8). Sin embargo, en pocos estudios se ha evaluado el potencial de estas herramientas de práctica mental de manera aislada. Hasta el momento, ningún estudio ha evaluado la influencia de la práctica mental de manera aislada sobre el control motor cervical.  Estas intervenciones no suponen ningún coste, ya que se basan tanto en imaginar, como en observar acciones de manera repetida, por lo que suponen avances dentro de las nuevas tecnologías sin requerir un equipo altamente sofisticado y sin personal altamente cualificado para su implementación (9).  Entre sus ventajas podemos destacar que no sobrecargan los tejidos blandos, pueden acelerar el retorno a la actividad física y son muy útiles para combinarlos con técnicas de fisioterapia tradicional. Además, no son terapias invasoras, y su aplicación es sencilla (13).  A nivel clínico, el control motor cervical es un aspecto muy relevante en la recuperación de los pacientes con dolor crónico cervical. Además, las mejoras en algunas variables como el reposicionamiento cervical parecen mejorar desde el primer momento de la aplicación de la práctica mental, aunque se necesitan más estudios para poder obtener conclusiones más sólidas y fiables. |
| CUALIFICACIÓN DEL EQUIPO INVESTIGADOR |
| 1. Personal que llevará a cabo las tareas con seres humanos y/o sus datos (duplicar las veces que sean necesarias)  - Nombre: Roy La Touche - NIF: - Vinculación: Director de la investigación - Nivel académico: Grado de Doctor (Ph. D.) - Tarea concreta en la investigación: Diseño de la investigación, análisis estadístico y redacción del manuscrito - Experiencia: Dirección de 9 tesis doctorales y publicación de más de 50 artículos científicos en revistas nacionales e internacionales con factor de impacto. Director del instituto de neurociencias y ciencias del movimiento (INCIMOV) y del grupo de investigación de Motion in Brains. - Nombre: Ferran Cuenca Martínez - NIF: - Vinculación: Investigador - Nivel académico: Grado de Máster (M. Sc.) - Tarea concreta en la investigación: Diseño de la investigación, llevar a cabo la intervención, análisis estadístico y redacción del manuscrito - Experiencia: Publicación de 7 artículos científicos en revistas internacionales con factor de impacto y miembro del grupo de investigación INCIMOV y Motion in Brains. - Nombre: Luis Suso Martí - NIF: - Vinculación: Investigador - Nivel académico: Grado de Máster (M. Sc.) - Tarea concreta en la investigación: Recogida de datos y redacción del manuscrito - Experiencia: Publicación de 6 artículos científicos en revistas internacionales con factor de impacto y miembro del grupo de investigación INCIMOV y Motion in Brains. |
| VALIDEZ CIENTÍFICA Y METODOLÓGICA |
| 1. Finalidad del proyecto:   Investigación básica  Investigación aplicada a la prevención, profilaxis, diagnóstico o tratamiento de enfermedades, mala salud u otras anomalías o sus efectos en los seres humanos.  Investigación aplicada a la evaluación, detección, regulación o modificación de las condiciones fisiológicas en los seres humanos.  Investigación aplicada al desarrollo de productos tecnológicos  Investigación aplicada a la protección del medio natural en interés de la salud o el bienestar de los seres humanos  Enseñanza superior o la formación para la adquisición o mejora de las aptitudes profesionales.  Investigación médico legal  Otra finalidad (concretar) |
| 1. Hipótesis y objetivos.   Hipótesis:  La intervención a través de la observación de acciones, en combinación con la imaginería motora que engloban tareas de control motor cervical, provocará un mejor reclutamiento motor en sujetos asintomáticos que en sujetos con dolor de cuello crónico. Por tanto, habrá un mayor aprendizaje motor en el grupo de sujetos sanos que en el grupo de sujetos con dolor de cuello a través de la práctica mental basada en la combinación de observación de acciones e imaginería motora.  Objetivos:  Objetivo Principal: Evaluar en qué grupo de intervención, bien en el grupo de sujetos asintomáticos, o bien en el grupo de sujetos con dolor de cuello, habrá un mayor aprendizaje motor con respecto al control motor cervical a través de práctica mental basada en la combinación de imaginería motora y observación de acciones de gestos motores de la región cervical.  Objetivo secundario: Evaluar las posibles asociaciones entre la mejora del control motor cervical con mayores niveles de actividad física, mayor capacidad de imaginar, puntuación de medidas de auto-informe de variables psicosociales y la cronometría mental. |
| 1. Diseño metodológico: carácter cuantitativo o cualitativo, diseño de la investigación, modalidad de estudio, método de aleatorización y análisis previsto de los resultados.   El presente estudio es un estudio de cohortes prospectivo. El evaluador será cegado a la asignación de los sujetos participantes en los grupos de intervención.  El evaluador estará cegado con respecto al grupo que pertenecen los sujetos que evalúa. Los participantes pueden hablar con los investigadores, pero no podrán comentar nada con el evaluador, ya que éste no tiene que condicionarse por el grupo al que pertenezca el sujeto.  No existirá aleatorización debido al tipo de estudio, el cual, contará con un muestreo no probabilístico por conveniencia.  El análisis estadístico previsto de los resultados será llevado a cabo a través del software SPSS v. 23.0 (SPSS Inc., Chicago, IL, USA). Para el análisis de los datos, se utilizará un intervalo de confianza del 95%, considerándose el valor *p* < 0,05 como estadísticamente significativo. Se utilizará el test de Shapiro-Wilk para comprobar la distribución normal de los datos. Se usará el test ANOVA de un factor para comparar las medias de las variables demográficas y las variables de control entre los dos grupos. En adición a esto, también se utilizará el test ANOVA de medidas repetidas sobre las variables de interés para observar las comparaciones inter e intra-grupo con ajuste post-hoc de Bonferroni. El análisis de correlaciones entre las variables principales y secundarias se llevará a cabo mediante el análisis de correlaciones de Pearson. Finalmente, el tamaño del efecto (*d* de Cohen) se calculará para las variables principales. De acuerdo con el método Cohen, el efecto será considerado pequeño (0,20 a 0,49), medio (0,50 a 0,79) o grande (> 0,80). |
| 1. Sistema de selección de la población y muestra. Describir el proceso de reclutamiento de datos de carácter personal y/o sujetos de investigación.   Para el muestreo de los sujetos de investigación, se colocarán carteles informativos en las instalaciones del Centro Universitario de La Salle destinados a alumnos, personal docente y administrativo. Debido a la alta prevalencia del dolor de cuello en la población universitaria, y en el personal docente y administrativo, se considerará que este sistema de elección será suficiente para alcanzar el tamaño muestral requerido para la realización del estudio. |
| 1. ¿El reclutamiento de la muestra va a ser realizado por alguien ajeno al equipo investigador?   No.  Si. En este caso indicar el nombre y apellidos, así como su vinculación a la investigación |
| 1. Criterios para la inclusión/ exclusión de participantes en la investigación.   Criterios de inclusión (grupo de sujetos asintomáticos):   - Sujetos sanos con edades comprendidas entre 18 y 60 años. - No estar tomando medicación durante el periodo del estudio. - No tener ninguna condición dolorosa. - Sujetos sin historia previa de patología cardio-respiratoria. - Hablar y comprender el idioma español, siendo capaces de responder a preguntas verbales, cuestionarios y entender las instrucciones durante los procedimientos del estudio.   Criterios de inclusión (grupo de sujetos con dolor de cuello):   - Sujetos sanos con edades comprendidas entre 18 y 60 años. - Sujetos con más de 3 meses de dolor de cuello en el último año   Criterios de exclusión (para ambos grupos):   - Sujetos que presenten patología sistémica, cardio-respiratoria, del Sistema Nervioso Central o enfermedad reumática. - Sujetos menores de edad. - Sujetos que no se encuentren en pleno uso de sus facultades mentales para poder realizar la intervención del estudio.   Eliminación:   - Falta de asistencia del sujeto a la cita prevista para la realización del estudio. - Aparición de trastornos psicológicos graves durante la realización del estudio.   ¿Se realiza con personas vulnerables? No  Embarazadas  Menores  Personas con discapacidad  Personas privadas de libertad |
| 1. Intervenciones en seres humanos. Especificar a continuación.   Entrevistas, encuestas  Fotos, grabaciones o similar  Pruebas físicas  Pruebas psíquicas  Otras |
| 1. Describir el procedimiento-intervención concreto sobre seres humanos que se va a llevar a cabo.   Grupo de Intervención 1: El grupo 1, formado por sujetos asintomáticos, realizará una intervención basada en la combinación de imaginería motora y observación de acciones de ejercicios de estabilización cervical.  Grupo de Intervención 2: El grupo 2, formado por sujetos con dolor de cuello, realizará también una intervención basada en la combinación de imaginería motora y observación de acciones de ejercicios de estabilización cervical.  En primer lugar se realizará la evaluación de las medidas basales tales como el nivel de actividad física, el registro de auto-informes, la cronometría mental, y la capacidad de generar imágenes mentales motoras, para poder ver la capacidad de imaginar de los participantes y su nivel de actividad física cotidiana. Posteriormente se realizará una evaluación pre-intervención del control motor cervical y tras la realización de la intervención, de aproximadamente 10 minutos de duración, se llevará a cabo inmediatamente la medición post-intervención. De esta forma se podrá evaluar el aprendizaje motor en la región cervical. El periodo de evaluación pre-intervención conllevará un tiempo aproximado de 25 minutos y la evaluación post-intervención de 10 minutos. Es por lo tanto que el tiempo total de intervención y evaluaciones tendrá una duración aproximada de 45 minutos. |
| 1. Describir cómo se realiza la selección de las personas que serán parte del grupo control.   Debido al diseño de investigación, no habrá grupo control en el presente estudio.  ¿Se les priva de una intervención de la que ya se conoce la eficacia de sus resultados?  Sí  No |
| 1. Intervención en el grupo control   Describir todas las pruebas o intervenciones a las que se va a someter al sujeto de estudio.  En el caso de utilizar placebo describir sus características  En el presente estudio no habrá un grupo control, ergo no procede.  Todas las intervenciones son las siguientes:   - Grupo de Intervención 1: El grupo 1, formado por sujetos asintomáticos, realizará una intervención basada en la combinación de imaginería motora y observación de acciones de ejercicios de estabilización cervical. - Grupo de Intervención 2: El grupo 2, formado por sujetos con dolor de cuello, realizará también una intervención basada en la combinación de imaginería motora y observación de acciones de ejercicios de estabilización cervical. |
| 1. Instrumentos utilizados para la obtención y recolección de información relacionada con el objeto de estudio. Describir todas las pruebas a las que se va a someter al sujeto de estudio para obtener sus datos especificando:   En primer lugar, todas las variables a describir pueden tener una influencia en la capacidad de aprendizaje motor y es por ello que se evalúan. Toda ellas se utilizarán de medidas basales las cuáles serán recogidas antes de la intervención.  Cuestionario Internacional de Actividad Física (IPAQ)  El cuestionario IPAQ es muy importante recoger debido a que amplia literatura ha demostrado que las personas con mayores niveles de actividad física presentan una capacidad mayor de generar imágenes mentales motora, ergo, es de alta importancia recolectar datos sobre los niveles de actividad física. Se utilizará el cuestionario IPAQ validado y traducido al español. El objetivo del cuestionario IPAQ es obtener datos relacionados con actividad física relacionada con salud. Se utilizará la versión larga formada por 5 objetivos de actividad evaluados independientemente que incluyen preguntas acerca de la actividad física relacionada con el trabajo, con el transporte, con el trabajo en el hogar, con el tiempo libre y finalmente con el tiempo dedicado a estar sentado. Los resultados finales sugieren que estas medidas tienen aceptables propiedades de medición y son apropiadas para estudios nacionales poblacionales de prevalencia de participación en actividad física (17).   - Lugar y persona encargada de realizarlas: Laboratorio del Centro Superior de Estudios Universitarios La Salle. Lo llevará a cabo Luis Suso Martí. - Duración: 2 minutos - Frecuencia: 1 sola vez - Posibles molestias o daños: ninguno - Otras cuestiones para tener en cuenta: no procede   Cuestionario de Imagen del Movimiento (MIQ-R)  El MIQ-R es un cuestionario con el objetivo de evaluar la capacidad de imaginación de los sujetos, el cual, está formado por ocho ítems, cuatro para una escala visual, y los otros cuatro para una escala cinestésica.  La puntuación de cada ítem del MIQ-R se efectúa en una escala de 7 puntos, donde el 1 indica “muy difícil de ver/sentir”, y el 7 indica “muy fácil de ver/sentir”.  La versión española del cuestionario MIQ-R tiene una consistencia interna alta y se correlaciona significativamente con otras medidas de imaginación de un movimiento u acción, así como con otras medidas que evalúan la claridad y agudeza de la capacidad de imaginar (18).   - Lugar y persona encargada de realizarlas: Laboratorio del Centro Superior de Estudios Universitarios La Salle. Lo llevará a cabo Luis Suso Martí. - Duración: 5 minutos - Frecuencia: 1 sola vez - Posibles molestias o daños: ninguno - Otras cuestiones para tener en cuenta: no procede     Cronometría mental  Utilizando un cronómetro, se registró el tiempo dedicado a realizar cada tarea del MIQ-R. El tiempo registrado corresponde al intervalo entre el comando para iniciar la tarea, dado por el evaluador, y la respuesta verbal de conclusión de la tarea, dada por el sujeto. La cronometría mental es una medida fiable y que ha sido utilizada ampliamente para recoger medidas objetivas de la capacidad de imaginar (19).   - Lugar y persona encargada de realizarlas: Laboratorio del Centro Superior de Estudios Universitarios La Salle. Lo llevará a cabo Luis Suso Martí. - Duración: 6 minutos - Frecuencia: 1 sola vez - Posibles molestias o daños: ninguno - Otras cuestiones para tener en cuenta: no procede   Control motor cervical  El control motor cervical se evaluará a través del reposicionamiento articular denominado “joint position error” mediante el aparato *Motion Guidance*. Este aparato, es un láser que se sujeta a la región craneocervical a través de gomas elásticas. Se pide al participante que mantenga un punto fijo mientras está sentado, posteriormente, se le pide que realice un movimiento y vuelva a su posición inicial. Se mide la distancia que separa el punto inicial del punto final medido en centímetros. Es una forma válida y fiable para la valoración sensomotora de la región cervical. Los parámetros de normalidad oscilan entre 3,81 centímetros y 5,08 centímetros. La confianza intra e inter-confiabilidad de este test es de 0.98 y 0.97 respectivamente, índice de correlación intra-clase es mayor a 0.62 (20).   - Lugar y persona encargada de realizarlas: Laboratorio del Centro Superior de Estudios Universitarios La Salle. Lo llevará a cabo Luis Suso Martí. - Duración: 1 minuto - Frecuencia: 2 veces - Posibles molestias o daños: ninguno - Otras cuestiones para tener en cuenta: no procede   Niveles de catastrofismo ante el dolor  El nivel de Catastrofismo se medirá a través de un auto-registro, mediante la Escala de Catastrofismo ante el Dolor, el cual, consta de 13 ítems que describen diferentes pensamientos y sentimientos asociados al dolor. La escala tiene 5 grados, siendo 0 nada en absoluto, 1 un poco, 2 moderadamente, 3 mucho y 4 todo el tiempo. El sujeto deberá marcar su situación en cada caso (21).   - Lugar y persona encargada de realizarlas: Laboratorio del Centro Superior de Estudios Universitarios La Salle. Lo llevará a cabo Luis Suso Martí. - Duración: entre 1 y 2 minutos - Frecuencia: 1 vez - Posibles molestias o daños: ninguno - Otras cuestiones para tener en cuenta: no procede     Miedo al movimiento o Kinesiofobia  El miedo al movimiento o Kinesiofobia se cuantificará a través de la versión española de la Escala Tampa de Kinesiophobia de manera auto-registrada. Este instrumento demuestra fiabilidad y validez para evaluar el miedo al movimiento, siendo más breve que la versión extendida de la escala. La puntuación total oscila entre 11 y 44 puntos, habiendo mayor kinesiofobia cuanto mayor puntaje en la escala (22).   - Lugar y persona encargada de realizarlas: Laboratorio del Centro Superior de Estudios Universitarios La Salle. Lo llevará a cabo Luis Suso Martí. - Duración: 2 minutos - Frecuencia: 1 veces - Posibles molestias o daños: ninguno - Otras cuestiones para tener en cuenta: no procede |
| 1. Plan de atención especializada derivada de los beneficios de la investigación tras la realización del proyecto, acceso al mismo de los participantes en la investigación de los participantes del grupo control.   Especificar:   - Intervención concreta que se prevea - Lugar - Tiempos   No procede, no habrá grupo control. |
| 1. ¿En el proyecto se utiliza un programa informático?   Sí  No  ¿Se ha consultado a la AEMPS (Agencia Española del Medicamento y Producto Sanitario) para saber si el programa que se va a utilizar se considera un “producto activo? No procede, el programa es un programa estadístico para el análisis de los resultados  Sí  No  ¿Presenta la consulta a la AEMPS?  Sí  No |
| 1. ¿Utiliza en el proyecto algún producto sanitario?   Sí  No  ¿Tienen marcado CE? No procede  Sí  No  ¿El producto sanitario se utiliza según las indicaciones del marcado CE? No procede  Sí  No  ¿Aporta el marcado CE? No procede  Sí  No |
| ASPECTOS ÉTICOS ESPECÍFICOS |
| **PONDERACIÓN BENEFICIOS/RIESGOS** |
| 1. Señale a continuación cualquier tipo de riesgo y /o molestia debidos a las pruebas que se realizan para el proyecto y la previsión en caso de acontecimientos adversos.   Riesgo físico  Riesgo psicológico (por ejemplo, un cuestionario puede representar un riesgo si se refiere a eventos traumáticos o especialmente estresante)  Riesgo social (por ejemplo, si el conocimiento de la información expresada puede llevar a enfrentar riesgo de estigmatización o discriminación)  Intromisión en la intimidad  Uso de su tiempo  Otras (describir)  No se prevé ningún riesgo derivado de imaginar u observar ejercicios de estabilización cervical. |
| 1. ¿Cómo valoraría el balance riesgo/beneficio?   Riesgo mínimo: aquel que no supera en probabilidad o magnitud al que cabría esperar en la actividad cotidiana, incluyendo exámenes físicos o psicológicos rutinarios.  Riesgo superior al mínimo: aquel que se produce cuando la posibilidad de daño físico o psicológico o relacionado con la invasión de privacidad o confidencialidad es mayor que el esperado en la actividad cotidiana. |
| 1. Previsión para evaluar y controlar eventualidades que pudieran suponer consecuencias para la salud presente y futura de los participantes en la investigación y/o de otras personas que pudieran verse afectadas por la investigación o sus resultados. (Medidas de seguridad en el procedimiento de la investigación)   No se prevén eventualidades que pudieran suponer consecuencias para la salud presente y futura de los participantes ante observar e imaginar gestos de las manos. En la investigación, no se realizará ninguna intervención que pueda suponer riesgos en la salud de los participantes. No obstante, los procedimientos que se llevarán a cabo se aplicarán en un entorno controlado para que el sujeto esté relajado y pueda desarrollar las actividades pautadas. |
| 1. Procedimientos para la compensación por daños que surjan en el contexto del proyecto de investigación   Intervención especializada  Lugar  Tiempo  Seguro de responsabilidad civil.  Durante la realización del estudio, se espera no causar ningún daño a los participantes que participen en el mismo. En caso de que se produzca algún daño derivado de este, se abordará de forma individualizada valorando la gravedad de los mismos e influencia que ha tenido el estudio para el agravamiento del estado del sujeto, y, en consecuencia se consensuará una forma de compensación que en ningún caso será de tipo económica. No obstante, el investigador principal está cubierto por un seguro de responsabilidad civil del Colegio de Fisioterapeutas. |
| **SOLICITUD DE CONSENTIMIENTO** |
| 1. Consentimiento informado para los participantes en la investigación (adjuntar modelo a la petición).   Para personas capaces de consentir.  Para el representante legal de la persona no capaz de consentir.  Para grabaciones o fotografías. |
| 1. Tarea para la que se solicita el consentimiento:   Para realizar a la persona participante intervenciones invasivas  Para realizar a la persona participante intervenciones no invasivas  Para uso de las muestras y/o los datos de la persona participante en el proyecto actual  Para almacenamiento y uso de las muestras y/o los datos en proyectos posteriores  Para cesión |
| 1. En el caso de que la población seleccionada sea de personas vulnerables indicar la especial protección de sus derechos y bienestar:   No procede. No habrá tal caso.  La investigación no puede realizarse igual con sujetos menos vulnerables.  Los riesgos no excederán aquellos asociados con exámenes médicos de rutina para ellos.  Si no son capaces de consentir su asentimiento será recogido a partir de los 12 años.  Se solicitará el permiso a sus representantes legales u otros apropiados. |
| 1. Describir brevemente el proceso de presentación del proyecto y el de obtención de consentimiento del sujeto fuente y/o su representante legal: quién (persona encargada/s), cómo (conversación, vídeo, folleto informativo, etc.), dónde (lugar de información y firma de documentos) y cuándo se realizan (momento en el que se lleva a cabo)   En relación al proceso de presentación del proyecto y el de obtención del consentimiento del sujeto fuente, el investigador 1, Ferran Cuenca Martínez, será la persona encargada de llevarlo a cabo mediante una conversación y un folleto informativo. Tras ello, en el Laboratorio del Centro Superior de Estudios Universitarios La Salle, se llevará a cabo la firma de documentos y se llevará a cabo al inicio del estudio, y por tanto, antes de empezar la valoración inicial así como cualquier intervención. |
| - - Método para asegurar el respeto a la vida privada de los participantes en la investigación y de la confidencialidad de los datos personales. ¿Cómo se han obtenido los datos de carácter personal?   Obtenidos expresamente para este proyecto con intervención del sujeto de estudio  Procedentes de datos recogidos para otro proceso o actividad (historia clínica, bases de datos estudio sociológico, de grupo etc.)  Se garantiza la confidencialidad, el tratamiento, la comunicación y la cesión de los datos de carácter personal de todos los sujetos participantes se ajustará a lo dispuesto según la legislación sobre protección de datos vigente en España (Ley de Protección de Datos de Carácter Personal, 03/2018, de 5 de diciembre). |
| 1. Información obligatoria mínima   ¿Se ha incluido la siguiente información obligatoria en el documento de consentimiento?  Sí  No  Utilizar los siguientes puntos como lista de chequeo:   - Identificación del IP (apellidos y nombre). - Identificación del proyecto:   - Título y financiación si procede   - Descripción del proyecto     - Objetivos/beneficios esperados     - Duración   - Lugar de realización   - Método - Identificación de la persona que informa (apellidos, nombre) - Identificación de la persona que presta el consentimiento (apellidos, nombre) - Fechas y firmas de   - Participante si es mayor de edad   - Representante legal si es menor de edad o incapaz - Persona encargada de recabar el consentimiento.   En el consentimiento informado se encuentra toda la información obligatoria mínima anteriormente citada. |
| 1. Información sobre las intervenciones:   ¿Se describen y explican claramente las pruebas o intervenciones a las que va a ser sometido el sujeto participante?  Sí  No  Utilice los puntos siguientes como lista de chequeo:   - Tipo de prueba o intervención (p. ej. encuesta o tipo de prueba) - Descripción de la prueba o intervención y objetivo de esta.   - Número de veces que se va a realizar, fechas y plazos.   - Si se va a contactar con él/ella con posterioridad   - Descripción de riesgos y/o molestias y medidas para minimizarlas (incluido seguro si procede) - Se ofrece aclarar dudas y más información y forma de contacto.   Se describen y explican claramente las pruebas o intervenciones a las que va a ser sometido el sujeto participante. |
| 1. Cláusula de voluntariedad   ¿Se informa de que la participación en el proyecto es voluntaria y de que la negativa a hacerlo no supondrá́ ningún perjuicio o medida en su contra?  Sí  No |
| 1. Derecho de revocación   ¿Se informa de que el/la participante puede dejar el estudio en cualquier momento sin que eso le suponga ningún perjuicio o medida en su contra?  Sí  No  Utilice los siguientes puntos como lista de chequeo   - Se informa con quién debe contactar para hacerlo efectivo - Se informa de que tiene derecho a elegir qué debe hacerse con sus datos obtenidos hasta el momento - destrucción o anonimización - conservación datos de investigación obtenidos hasta ese momento   Todos los puntos se encuentran integrados explícitamente en el consentimiento informado con respecto al derecho de revocación. |
| 1. Derecho a decidir sobre el destino de los datos   ¿Se informa al participante sobre lo que se va a hacer con sus datos una vez finalizado el estudio y se le da a elegir su destino?  Sí  No  Utilice los siguientes puntos como lista de chequeo   - Destrucción - Anonimización para uso posterior. - Conservación de los datos para investigaciones relacionadas con la inicialmente propuesta. - Otro destino   Se informa al participante sobre lo que se va a hacer con sus datos una vez finalizado el estudio y se le da a elegir su destino. |
| 1. Cláusula de protección de datos   ¿Se informa al participante sobre todo lo relacionado con el uso de sus datos de carácter personal? (Cláusula de protección de datos)  Sí  No  Utilice los siguientes puntos como lista de chequeo.  Información mínima obligatoria que debe incluir:   - Identidad y datos de contacto del Responsable del tratamiento - Datos de contacto del Delegado de Protección de Datos - Finalidad del fichero (objeto de la investigación) - La categoría de datos personales que se traten - Cesiones previstas si las hubiera, condiciones de estas: datos personales, anonimizados o disociados; y destinatarios o categorías de destinatarios - Ante quién ejercer los derechos de acceso, rectificación, supresión y portabilidad de los datos, así como limitación y oposición al tratamiento - El carácter obligatorio o facultativo de las respuestas a las preguntas que le sean planteadas - Plazo durante el cual se conservarán los datos personales o, en su defecto, los criterios utilizados para determinar este plazo - El derecho a presentar una reclamación ante una autoridad de control - Si se recaban datos personales del/la interesado/a de fuentes distintas a él mismo/a. En caso afirmativo, habrá que informar, además de lo anterior, de la fuente de la que proceden los datos personales.   Se informa al participante sobre todo lo relacionado con el uso de sus datos de carácter personal. |
| 1. Derecho a conocer los resultados   ¿Se informa al sujeto participante de cómo puede conocer los resultados de la investigación?  Utilice los siguientes puntos como lista chequeo.  Sí  No   - Resultados generales (publicaciones…) - Derecho a no saber - Información a familiares   ¿Cómo se va a realizar la comunicación de los resultados en el caso de ser necesario?  En mano  Vía email, de ser así ¿se encriptan los datos?  Otras, especificar  Solo en el caso de ser necesario, se mandará una hoja informativa por correo a los participantes comunicando los resultados de la investigación, su resultado final y la fecha en la que serán expuestos dichos resultados con los datos encriptados. La criptografía se utiliza para enviar mensajes confidenciales cifrando el contenido a través de una línea de comunicación para que los sujetos puedan desencriptarlo. Se utilizará un sistema de criptografía simétrica o de clave privada donde las dos partes serán conocedoras de dicha claves para poder tener acceso a su información en caso de desearlo. Se llevará a cabo desde el primer momento para evitar la fuga de datos personales. |
| **PROTECCIÓN DE DATOS DE CARÁCTER PERSONAL Y DEBER DE CONFIDENCIALIDAD (DERECHO A LA CONFIDENCIALIDAD)** |
| 1. Método para el tratamiento de la información que pudiera generarse durante la investigación.   ¿Se van a guardar datos de carácter personal en un fichero?  Sí  No |
| 1. ¿Es estrictamente necesario el tratamiento de datos de carácter personal para la investigación realizada?   Sí  Puede llevarse a cabo con datos anonimizados |
| 1. ¿En qué soporte se guardarán los datos de carácter personal recogidos?   En soporte Microsoft Excel con una contraseña cifrada garantizando el acceso exclusivamente al equipo investigador |
| 1. Características del fichero. ¿qué sistema se va a utilizar para el tratamiento de los datos de carácter personal?   Los datos primarios o sin procesar serán guardados por los investigadores bajo llave en un archivo mientras que los datos procesados, serán guardados en un documento encriptado donde solamente los investigadores de esta investigación podrán tener acceso previo conocimiento de la clave provista.  Manual  Automatizado  Mixto  Manual con tratamiento automatizado con datos disociados |
| 1. ¿Existe personal externo al CSEULS, incluido el de empresas proveedores de sistemas, que vaya a tener acceso a los datos personales objeto de la investigación? En caso afirmativo, indique brevemente su identidad y la finalidad del acceso.   No. No procede. |
| 1. Seguridad del fichero: indique brevemente las principales medidas de seguridad que va a adoptar sobre los datos para evitar la identificación del sujeto fuente de los datos y preservar la confidencialidad.   Los datos recogidos para el estudio estarán identificados mediante un código (número de sujeto). Por lo tanto, su identidad no será revelada a ninguna persona.  No se transmitirán a terceros, a excepción del propio grupo de investigación Motion in Brains, ni a otros países los datos recogidos.  El acceso a la información personal quedará restringido a los investigadores, al Comité Ético de Investigación Clínica y personal autorizado por el investigador, cuando lo precisen para comprobar los datos y procedimientos del estudio, pero siempre manteniendo la confidencialidad de los mismos de acuerdo a la legislación vigente.  Los datos necesarios estarán guardados en nuestra base de datos encriptada bajo una contraseña segura que solo se podrán disponer de ellos en un equipo informático privado del investigador principal en la Facultad de Ciencias de la Salud del Centro Universitario La Salle. |
| 1. ¿Se recoge y conserva información que debe ser conservado en un fichero que contenga categorías de datos especialmente protegidos?  - Información médica: datos relativos a la salud, datos genéticos, datos biométricos.   Si, mientras sea necesario para el presente proyecto. |
| 1. Destino de los datos de carácter personal.    - ¿Qué se va a hacer con los datos de carácter personal una vez que ya no sean necesarios para este proyecto?   Destrucción  Conservación   - - Periodo de conservación de los datos   No procede   - - Lugar y condiciones de conservación   En un armario bajo llave en el despacho del investigador principal en la Facultad de Ciencias de la Salud del Centro Universitario La Salle. |
| **OTRAS CUESTIONES ÉTICAS** |
| 1. Detalle de los emolumentos y compensaciones propuestos por participar en la investigación. En el caso de que exista algún tipo de incentivo o compensación indicar su naturaleza.   La participación en este estudio es voluntaria y, por tanto, los participantes no recibirán ninguna compensación económica. |
| 1. Detalle de las circunstancias que podrían conducir a conflicto de interés y que podrían afectar al juicio independiente de los investigadores.   Debido a la ausencia de inversores y empresas que financien el presente estudio, no habrá ningún conflicto de interés. |
| 1. Detalle de cualquier uso futuro potencial, incluyendo el comercial, de los resultados de investigación, de otros datos recogidos durante el desarrollo del proyecto, o de muestras biológicas.   Se espera que los datos obtenidos con la realización de este proyecto puedan tener uso potencial en el ámbito clínico y en la práctica deportiva, donde el aprendizaje motor es un aspecto ampliamente relevante y clave, así como en aquellas prácticas que requieran un aprendizaje motor tales como los pacientes con falta de control motor cervical debido a la presencia de una condición dolorosa, o recuperación tras inmovilización prolongada por desuso o cirugía. Los investigadores del estudio creen que este estudio puede tener una repercusión en el mundo de la Fisioterapia y del deporte, pero es posible que esto no ocurra. |
| 1. Detalle de la existencia de cualquier compensación, pago o bienes que sean proporcionados al investigador o su institución a cambio de la investigación.   El presente proyecto no tiene ningún tipo de interés económico, ni hay empresas o entidades que financien el estudio. Por lo tanto, al no haber ninguna fuente de recursos y ser un estudio voluntario para los sujetos que participen en él, no hay presente ningún emolumento para los investigadores responsables del mismo. |
| 1. Otras consideraciones éticas, que los autores de la investigación consideren oportuno indicar   Durante la realización del estudio, los investigadores se comprometen a trabajar con la máxima diligencia posible sin coaccionar a los sujetos participantes del mismo. Por otra parte, se dejará a los sujetos total libertad para exponer las posibles preguntas e inquietudes sobre las características teóricas y prácticas implicadas. Asimismo, los investigadores intentarán resolver las dudas que surjan con el máximo rigor científico, adaptando las respuestas al nivel de compresión de cada uno de los sujetos. |
| 1. Bibliografía utilizada.   1. Decety J. The neurophysiological basis of motor imagery. Behav Brain Res. 1996;77(1-2):45–52.  2. Mulder T. Motor imagery and action observation: cognitive tools for rehabilitation. J Neural Transm. 2007;114(10):1265–78.  3. Calmels C, Holmes P, Lopez E, Naman V. Chronometric Comparison of Actual and Imaged Complex Movement Patterns. J Mot Behav. 2006;38(5):339–48.  4. Guillot A, Collet C. Construction of the Motor Imagery Integrative Model in Sport: a review and theoretical investigation of motor imagery use. Int Rev Sport Exerc Psychol. 2008;1(1):31–44.  5. Ridderinkhof KR, Brass M. How Kinesthetic Motor Imagery works: A predictive-processing theory of visualization in sports and motor expertise. J Physiol. 2015;109(1-3):53–63.  6. Feltz DL, Landers DM. The Effects of Mental Practice on Motor Skill Learning and Performance: A Meta-analysis. J Sport Psychol. 1983 Mar;5(1):25–57.  7. Sakamoto M, Muraoka T, Mizuguchi N, Kanosue K. Combining observation and imagery of an action enhances human corticospinal excitability. Neurosci Res. 2009;65(1):23–7.  8. Taube W, Mouthon M, Leukel C, Hoogewoud H-M, Annoni J-M, Keller M. Brain activity during observation and motor imagery of different balance tasks: An fMRI study. Cortex. 2015;64:102–14.  9. Vogt S, Rienzo F Di, Collet C, Collins A, Guillot A. Multiple roles of motor imagery during action observation. Front Hum Neurosci. Frontiers; 2013;7:807.  10. Gonzalez-Rosa JJ, Natali F, Tettamanti A, Cursi M, Velikova S, Comi G, et al. Action observation and motor imagery in performance of complex movements: Evidence from EEG and kinematics analysis. Behav Brain Res. 2015;281:290–300.  11. Roman-Viñas B, Serra-Majem L, Hagströmer M, Ribas-Barba L, Sjöström M, Segura-Cardona R. International Physical Activity Questionnaire: Reliability and validity in a Spanish population. Eur J Sport Sci. 2010;10(5):297–304.  12. Campos A, González MÁ. Spanish version of the revised movement image questionnaire (MIQ-R): Psychometric properties and validation. 2010;19(2):265–75.  13. Guillot A, Collet C. Duration of Mentally Simulated Movement: A Review. J Mot Behav [Internet]. 2005;37(1):10–20.  14. Malouin F, Richards CL, Durand A, Doyon J. Reliability of Mental Chronometry for Assessing Motor Imagery Ability After Stroke. Arch Phys Med Rehabil. 2008;89(2):311–9.  15. Williams SE, Guillot A, Di Rienzo F, Cumming J. Comparing self-report and mental chronometry measures of motor imagery ability. Eur J Sport Sci. 2015;15(8):703–11.  16. Spruijt S, Jongsma MLA, van der Kamp J, Steenbergen B. Predictive models to determine imagery strategies employed by children to judge hand laterality. PLoS One. 2015;10(5):e0126568.  17. Roman-Viñas B, Serra-Majem L, Hagströmer M, Ribas-Barba L, Sjöström M, Segura-Cardona R. International Physical Activity Questionnaire: Reliability and validity in a Spanish population. Eur J Sport Sci. 2010;10(5):297–304.  18. Campos A, Gonzalez MA. Spanish Version of the Revised Movement Image Questionnaire (Miq-R): Psychometric Properties and Validation. Rev Psicol Del Deport. 2010;19(2):265–75.  19. Malouin F, Richards CL, Durand A, Doyon J. Reliability of Mental Chronometry for Assessing Motor Imagery Ability After Stroke. Arch Phys Med Rehabil [Internet]. 2008 Feb [cited 2017 Jul 12];89(2):311–9.  20. Revel, M, Andre-Deshays, C, and Minguet, M. Cervicocephalic kinesthetic sensibility in patients with cervical pain. *Arch Phys Med Rehabil* 72: 288–91, 1991.  21. Olmedilla Zafra, A, Ortega Toro, E, and Abenza Cano, L. Validación de la escala de catastrofismo ante el dolor (Pain Catastrophizing Scale) en deportistas españoles. *Cuad Psicol del Deport* 13: 83–94, 2013.  22. Gómez-Pérez, L, López-Martínez, AE, and Ruiz-Párraga, GT. Psychometric Properties of the Spanish Version of the Tampa Scale for Kinesiophobia (TSK). *J Pain* 12: 425–35, 2011. |
| Anexos: documentación aportada.  Consentimiento informado.  Consentimiento audiovisual.  Permiso/Declaración jurada y otra documentación requerida para personas no capaces consentir.  CV de los investigadores.  Permiso o aceptación de un centro colaborador para recoger en su sede las muestras necesarias o datos de la historia clínica de sus pacientes.  Permiso o aceptación de un centro externo colaborador para realizar en sus instalaciones los procedimientos de intervención.  Contrato de acceso a datos de carácter personal con el centro colaborador.  Consulta a la AEMPS en el caso de utilizar un programa informático.  Marcado CE en el caso de utilizar un producto sanitario.  Otros: |
| **TRATAMIENTO DE DATOS PERSONALES EN VIRTUD DE ESTE PROTOCOLO** |
| Los datos personales que se faciliten en el presente protocolo (Investigador Principal, investigadores, persona responsables en centros externos, entre otros) serán tratados por parte de CSEULS con la finalidad de gestionar el proyecto de investigación. El tratamiento de datos se encuentra legitimado por la existencia de una relación contractual. Los datos personales únicamente serán comunicados a instituciones o entidades donde se realice la investigación y podrán ser accedidos por terceros que provean servicios a CSEULS y que sean necesarios para el proyecto (como, por ejemplo, proveedores de soluciones de almacenamiento). Al margen de lo anterior, únicamente se cederán datos a terceros por obligación legal.  Los datos cedidos a terceros, si se ceden serán datos totalmente codificados.  Los datos personales serán conservados durante todo el tiempo que dure la relación contractual. Los titulares de los datos personales pueden ejercitar sus derechos de acceso, rectificación, supresión y portabilidad de los datos, así como de oposición y limitación del tratamiento, a través de correo postal a calle Ganímedes, 11, 28023 Aravaca (Madrid) o de correo electrónico a [lopd@lasallecampus.es](mailto:lopd@lasallecampus.es). Puede contactar también para asuntos relacionados con el tratamiento de sus datos personales con el Delegado de Protección de Datos de CSEULS a través de correo electrónico en [dpo@castaneda-abogados.com](mailto:dpo@castaneda-abogados.com). Adicionalmente, puede recabar la tutela de la Agencia Española de Protección de Datos, cuyos datos de contacto se ofrecen a través de [www.aepd.es](http://www.aepd.es) |
